# Morbidity before and after the Diagnosis of Hyperthyroidism: A Nationwide Register-Based Study

**DOI:** 10.1371/journal.pone.0066711

**Published:** 2013-06-20

**Authors:** Frans Brandt, Marianne Thvilum, Dorthe Almind, Kaare Christensen, Anders Green, Laszlo Hegedüs, Thomas Heiberg Brix

**Affiliations:** 1 Department of Endocrinology and Metabolism, Odense University Hospital, Odense, Denmark; 2 The Danish Aging Research Center and The Danish Twin Registry, University of Southern Denmark, Odense, Denmark; 3 Department of Clinical Genetics, Odense University Hospital, Odense, Denmark; 4 Department of Clinical Biochemistry and Pharmacology, Odense University Hospital, Odense, Denmark; 5 Odense Patient Data Exploratory Network, Institute of Clinical Research, University of Southern Denmark, Odense, Denmark; IPATIMUP/Faculty of Medicine of the University of Porto, Portugal

## Abstract

**Background:**

Hyperthyroidism has been linked with different morbidities, like atrial fibrillation, stroke and diabetes mellitus. However, our knowledge regarding the extent and temporal relation between hyperthyroidism and other diseases is fragmented. Here, we aimed at evaluating various morbidities before and after the diagnosis of hyperthyroidism.

**Methods:**

Observational cohort study. From nationwide Danish health registers 2631 hyperthyroid singletons and 375 twin pairs discordant for hyperthyroidism were identified and followed for an average of 6 years (range 0–13). Data on the occurrence of cardiovascular diseases, lung diseases, diabetes mellitus, rheumatic diseases and malignant diseases was obtained by person-to-person record linkage with the National Danish Patient Register and/or the Danish National Prescription Registry (lung diseases and diabetes mellitus). Logistic and Cox regression models were used to assess the risk of morbidity before and after the diagnosis of hyperthyroidism, respectively. All Cox regression analyses were adjusted for the degree of co-morbidity preceding the diagnosis of hyperthyroidism, using the Charlson score.

**Results:**

Hyperthyroid individuals had a significantly higher risk of being diagnosed with cardiovascular diseases (odds ratio (OR) 1.65; 95% confidence interval (CI): 1.45–1.87), lung diseases (OR 1.53; 95% CI: 1.29–1.60), and diabetes mellitus (OR 1.43, 95% CI: 1.20–1.72), but not with malignant diseases (OR 1.16, 95% CI: 0.99–1.36) prior to the diagnosis of hyperthyroidism. After the diagnosis of hyperthyroidism, subjects had a significantly higher risk of being diagnosed with cardiovascular diseases (hazard ratio (HR) 1.34; 95% CI: 1.15–1.56), lung diseases (HR 1.28; 95% CI: 1.10–1.49), and diabetes mellitus (HR 1.46; 95% CI: 1.16–1.84), but not with rheumatic diseases (HR 1.39, 95% CI: 0.92–2.09) or malignant diseases (HR 1.18, 95% CI 0.97–1.42).

**Conclusions:**

We demonstrate a significantly increased burden of morbidity, both before and after the diagnosis of hyperthyroidism.

## Introduction

Next to diabetes mellitus (DM) and osteoporosis, hyperthyroidism is one of the most common endocrine disorders, with a lifetime risk of 2–5% [1]. Regardless of its cause, whether autoimmune or caused by autonomous nodules, the etiology is based on a complex interplay between genetic [2] and environmental factors, of which iodine intake and smoking habits are best documented [3], [4]. While thyroid hormones affect all organ systems, it is well accepted that the cardiovascular system is a major target of thyroid hormone action [5]. Short-term hyperthyroidism is characterized by a hyperdynamic cardiovascular state (high cardiac output with low systemic resistance) [6], while long-term exposure to thyroid hormone excess may lead to more severe changes, including pulmonary hypertension [7], diastolic dysfunction [8], and subsequent heart failure [9]. In addition, thyroid hormones have a direct effect on endothelial function [10] and coagulation [11]. Thus, it is no surprise that hyperthyroidism has been associated with an increased risk of cardiovascular diseases (CVD) [10], [12], [13]. On the other hand, diagnostic procedures and/or treatment of CVD may increase the risk of developing hyperthyroidism e.g. due to the use of iodine containing substances (i.e. x-ray contrast agents and amiodarone) [14]. Moreover, the interpretation of a potential association between hyperthyroidism and a diagnosis of CVD may be complicated by misclassification of hyperthyroidism due to non-thyroidal illness [15] or increased awareness of thyroid disease resulting in selection bias or confounding by indication [16]. Also genetic confounding could hamper the interpretation of data, since hyperthyroidism [17], CVD [18] as well as stroke [19] demonstrate familial and to some degree individual clustering. Clearly, the same reservations hold true for the observed associations between hyperthyroidism and rheumatic diseases (RD) [20], lung diseases (LD) [21], malignant diseases (MD) [22], as well as DM [23].

Despite these uncertainties regarding the observed associations between hyperthyroidism and morbidity, our recent studies provide robust evidence of an association between thyroid dysfunction, hyper- [24]–[26] as well as hypothyroidism [27], [28], and an overall increased mortality. Unfortunately, the current literature, based on underpowered studies [20], only allows speculation as to whether the increased mortality seen in hyperthyroidism is related to an increased disease frequency before or after the diagnosis of hyperthyroidism. Therefore, our aim has been to evaluate the temporal relation between hyperthyroidism and CVD, RD, LD, MD, and DM in a population-based study of 2631 hyperthyroid singletons and in 375 same-sex twin pairs discordant for hyperthyroidism.

## Materials and Methods

### Data Sources

The Danish Civil Registration System (DCRS) and The Danish Demographic Database (DDB) cover information on demographics, vital status, date of death and residence of all persons living or having lived in Denmark from 1968 [29]. From DCRS, we have identified a random 5% sample of all Danes representing the birth cohorts 1870–2001.

The Danish Twin Registry (DTR) is a nation-wide and population-based register, established in 1954. It comprises nearly 75,000 twin pairs born in Denmark from 1870 until 2001 [30]. All twin pairs are ascertained independently of zygosity and disease.

The Danish National Patient Registry (DNPR) includes registrations of all admissions to hospitals (both primary and secondary diagnoses) since January 1, 1977. Outpatient admissions have been registered separately since January 1, 1995 [29]. All registrations are according to the International Classification of Diseases (ICD). The validity of DNPR is high and misclassification of hyperthyroidism has been shown to occur in less than 2% of cases [31].

The Danish National Prescription Registry (DNPrR) provides information on all prescriptions of drugs dispensed from Danish pharmacies since 1995 [29]. Coding for medical products is according to the Anatomical Therapeutic Chemical (ATC) classification system. Besides the ATC code the register covers information on date of dispensing, strength, and quantity (in defined daily doses). In Denmark, the national health security system covers all inhabitants and partially reimburses drug expenses. Data from DNPrR are transmitted directly from the cash register in the pharmacy and used in the calculation (made on an individual level) of the expenses reimbursed.

All the databases described are accessible through Statistics Denmark [29]. DCRS is based on a unique 10 digit personal identification number (CPR-number) assigned to all persons living or having lived in Denmark. The CPR-number allows record-linkage between all the mentioned databases on an individual level.

### Diagnosis of Hyperthyroidism

Information on thyroid status was drawn from DNPR or DNPrR [29]. To be classified as having hyperthyroidism subjects should be recorded in at least one of these registers. In DNPR, hyperthyroidism was defined by ICD-8 codes 242.00–242.99 (1977–1994) and the ICD-10 codes E05–E05.9 (1995–2008). Graveś disease (GD) was defined by the ICD-8 codes 242.08, 242.09, 242.00 or 242.01 as well as by the ICD-10 code E05.0. Toxic nodular goiter (TNG) was defined by the ICD-8 code 242.19 and the ICD-10 codes E05.1 or E05.2. In DNPrR hyperthyroidism was defined by at least two dispensed prescriptions of anti-thyroid medication (ATC = H03B). Date for diagnosis of hyperthyroidism (index-date) was chosen as the first date of registration in either DNPR or DNPrR.

### Study Population

The study populations were identified from DCRS or DTR [29], [30]. In order to investigate incident cases - and to obtain the same observation frame in DCRS, DTR, DNPR, and DNPrR - only individuals over 18 years of age and diagnosed with hyperthyroidism after December 31, 1995 were included. In all, we identified 2631 singleton individuals with hyperthyroidism from the random 5% sample of the Danish singleton background population (n = 339481). From DNPR, 759 singletons were identified with GD and 466 with TNG. All singletons identified with hyperthyroidism were matched with four control subjects according to age and sex, following the principles of density sampling [32]. In addition, we identified 375 same-sex twin pairs discordant for hyperthyroidism. All participants were followed from the date of the hyperthyroid diagnoses until migration, death or December 31, 2008.

### Morbidity

The Charlson score (CS) accounts for 19 disease groups (myocardial infarction, heart failure, vascular disease, cerebrovascular disease, dementia, chronic lung disease, rheumatic disease, gastric ulcer, liver disease, diabetes mellitus without complications, diabetes mellitus with complications, hemiplegia, kidney disease, cancer, cancer with metastases, lymphoma, leukemia, liver failure and AIDS) by creating a weighted score on an individual level, to optimize the prediction of the one-year mortality risk within each disease category [33]. The CS was originally constructed to estimate one-year mortality in patients with breast cancer, but has subsequently been validated and used in different phenotypes including non-malignant diseases [34]. All the disease categories within the CS were identified from DNPR. As patients with DM and obstructive airways diseases are often diagnosed and treated solely in primary care, all users of anti-diabetics (ATC: A10) and drugs for obstructive airway diseases (ATC: R03) identified from DNPrR [29], were categorized with DM and LD, respectively. All relevant ATC-codes as well as ICD-8 and ICD-10 codes are shown in table 1.

**Table 1:** Definition of morbidity from data in the Danish National Patient Registry and the Danish National Prescription Registry.

Morbidity - outcome	ICD-8^a^	ICD-10^b^	ATC^c^
**Cardiovascular diseases**	344, 410, 427, 428, 430–438, 440–445 and 782	DI1, DI2, DI5–DI7, DG4 and DG8	_
**Lung diseases**	490–493 and 515–518	DJ4, DJ6, DJ8 and DJ9	R03
**Diabetes mellitus**	249 and 250	DE1	A10
**Rheumatic diseases**	135, 446, 712 and 734	DO8, DM0 and DM3	_
**Malignant diseases**	140–163, 170–174, 180–199 and 200–207	DC0–DC9	_
**Other diseases** d	290, 293, 530–534, 571, 573, 070, 456, 344,403–404, 580–584, 590, 593, 753, 792 and 079	DFO, DG3, DK2, DK7, DB1, DG8, DI1, DN0–DN1, DQ6 and DB2	_

a International Classification of Diseases 8.

b International Classification of Diseases 10.

c Anatomical Therapeutic Chemical classification system.

d Dementia, gastric ulcer, liver disease, hemiplegia, kidney disease, liver failure and AIDS.

The CS, prior to the diagnosis of hyperthyroidism was used in the adjustment of the morbidity risk in the analyses after the diagnosis of hyperthyroidism. For subjects with hyperthyroidism, the CS covers the time period from January 1, 1977 (start of DNPR) until the index-date. In controls, the CS covers the time period from the start of DNPR until the index-date of the corresponding case.

The overall outcomes, categorized into CVD, RD, LD, MD, DM and other diseases (dementia, gastric ulcer, liver disease, hemiplegia, kidney disease, liver failure and AIDS), were defined on the basis of the 19 disease groups covered by the CS (Table 1). For each individual the first date of possible registration in each of these groups was identified. Accordingly, we stratified for the first registration in each disease group before and after the date of the diagnosis of hyperthyroidism.

### Data Analysis

Group frequencies were compared with the Pearson X^2^ test, whereas group means and medians were compared by a t-test. In the case of paired comparisons the paired t-test was used.

The odds ratio (OR) for morbidity, prior to the diagnosis of hyperthyroidism, was evaluated in a logistic regression analysis adjusted for age and sex. The relationship between hyperthyroidism and morbidity, after the diagnosis of hyperthyroidism, was evaluated by the Cox regression model. Age was chosen as the underlying time variable. In both singleton cases and controls, person years of follow up were accumulated from the index-date in the case and were terminated at the date of diagnosis of morbidity in the relevant disease category, migration, death or end of follow-up (December 31, 2008), whichever came first. In all Cox analyses the variable “pair” was used as a stratum variable, fixing the baseline hazard within a matched pair, while at the same time allowing this baseline hazard to vary freely between pairs. Subsequently, all Cox regression analyses were adjusted for the degree of co-morbidity preceding the diagnosis of hyperthyroidism, using the CS.

Additionally, we performed intra-pair analyses of the twin population in which the hyperthyroid twin was matched with the corresponding euthyroid co-twin. After pooling all disease groups the differences in disease frequency were evaluated by conditional logistic regression analyses.

Significant differences were defined as a p-value below 0.05, using two-tailed tests. All analyses were conducted using STATA version 11.0 (2009; Stata Corporation, College Station, TX, USA).

## Results

### Baseline Characteristics of the Study Population

The baseline characteristics of singletons and twins, diagnosed with hyperthyroidism, as well as their controls are shown in table 2. The mean age of singletons and twins, at diagnosis of hyperthyroidism, was 61 and 56 years, respectively. Cases and controls were followed for an average of 6 years (range 0–13, standard deviation 3.4 years).

**Table 2:** Characteristics of the study populations.

	Singletons		Twins^a^	
	Cases	Controls	Cases	Controls
Number	2631	10524	375	375
Mean age (years)	67	67	64	64
Mean age at diagnosis (years)	61		56	
Females (%)	81	81	82	82
Mean follow-up (years)	6	6	6	6

a Twins from pairs discordant for hyperthyroidism.

### Overall Associations between Hyperthyroidism and Morbidity

The results from the overall association analyses of hyperthyroidism and morbidity are shown in table 3. Compared with the control population singletons with hyperthyroidism had a higher frequency of CVD, LD, DM as well as the group of other diseases. Stratification for the cause of hyperthyroidism yielded essentially similar results (data not shown). The only difference was that TNG was also positively associated with RD (cases 6%, controls 3%, p<0.01).

**Table 3:** Temporality and impact of co-morbidity in hyperthyroidism.

Disease category	Overall association			Odds ratio^b^	Hazard ratio^c^
	Cases^a^	Controls^a^	P-value		
**Cardiovascular disease**	**677 (26%)**	**1992 (19%)**	**<0.001**	**1.65 (1.23–1.87)**	**1.34 (1.15–1.56)**
**Lung disease**	**780 (30%)**	**2409 (23%)**	**<0.001**	**1.53 (1.29–1.60)**	**1.28 (1.10–1.49)**
**Diabetes mellitus**	**283 (11%)**	**795 (8%)**	**<0.001**	**1.43 (1.20–1.72)**	**1.46 (1.16–1.84)**
**Rheumatic disease**	123 (5%)	418 (4%)	0.100	**1.19 (1.05–1.46)**	1.39 (0.92–2.09)
Malignant disease	370 (14%)	1329 (13%)	0.050	1.16 (0.99–1.36)	1.18 (0.97–1.42)
**Other diseases** d	**361 (14%)**	**1040 (10%)**	**<0.001**	**1.49 (1.23–1.79)**	**1.47 (1.17–1.84)**

a Number of individuals with a first time hit of the respective disease category.

b Odds ratios before the diagnosis of hyperthyroidism.

c Hazard ratios after the diagnosis of hyperthyroidism, adjusted for the Charlson score.

d Dementia, gastric ulcer, liver disease, hemiplegia, kidney disease, liver failure and AIDS.

Data given in bold represent significant findings.

### Morbidity before the Diagnosis of Hyperthyroidism

Singletons with hyperthyroidism had an increased risk of CVD (OR 1.65; 95% confidence interval (CI): 1.23–1.87), RD (OR 1.19; 95% CI: 1.05–1.46), LD (OR 1.53; 95% CI: 1.29–1.60), DM (OR 1.43; 95% CI: 1.20–1.72), and other diseases (OR 1.49; 95% CI: 1.23–1.79), prior to the diagnosis of hyperthyroidism (Table 3). Evaluating the same disease categories, but censoring diagnoses made within 365 days prior to the diagnosis of hyperthyroidism in order to evaluate potential confounding by indication, yielded essentially similar results (data not shown). Stratification for the cause of hyperthyroidism did not significantly change these findings as both singletons with GD and TNG had an increased risk of CVD (OR_GD_ 1.44; 95% CI: 1.06–1.96, OR_TNG_ 2.20; 95% CI: 1.70–2.82), RD (OR_GD_ 1.68; 95% CI: 1.06–2.65, OR_TNG_ 2.39; 95% CI: 1.36–4.19), LD (OR_GD_ 1.55; 95% CI: 1.27–1.89, OR_TNG_ 1.38; 95% CI: 1.06–1.79), and DM (OR_GD_ 1.91; 95% CI: 1.34–2.73, OR_TNG_ 1.64; 95% CI: 1.06–2.52).

### Morbidity following the Diagnosis of Hyperthyroidism

The hazard ratios (HR) for morbidity after the diagnosis of hyperthyroidism are shown in table 3. Singletons with hyperthyroidism had a significantly increased risk of being diagnosed with CVD (HR 1.34; 95% CI: 1.15–1.56), LD (HR 1.28; 95% CI: 1.10–1.49), DM (HR 1.46; 95% CI: 1.16–1.84), and other diseases (HR 1.47; 95% CI: 1.17–1.84), following the diagnosis of hyperthyroidism. Except for CVD (HR 1.07; 95% CI: 0.89–1.29), and other diseases (HR 0.99; 95% CI 0.74–1.33), these results did not change significantly, when censoring diagnoses made within 365 days following the diagnosis of hyperthyroidism (HR_LD_ 1.30; 95% CI: 1.09–1.54, HR_DM_ 1.37; 95% CI: 1.06–1.78). After stratification for the cause of hyperthyroidism, both singletons with GD and TNG had a significantly higher risk of being diagnosed with CVD (HR_GD_ 1.93; 95% CI: 1.46–2.56, HR_TNG_ 1.72; 95% CI: 1.26–2.34) DM (HR_GD_ 1.76; 95% CI: 1.14–2.70, HR_TNG_ 1.62; 95% CI: 1.00–2.65), and other diseases (HR_GD_ 1.90; 95% CI: 1.17–3.07, HR_TNG_ 1.88; 95% CI: 1.18–2.99).

### Morbidity in Twins

Analyzing all twins together, there was a non-significantly increased morbidity in the hyperthyroid twins when compared to the euthyroid co-twins (OR 1.13; 95% CI: 0.82–1.56). This was not the case when analyzing monozygotic (MZ) twins separately (OR 0.78, 95% CI 0.42–1.45). Due to the lack of power further subdivision, according to phenotype (GD or TNG) or morbidity groups, was not meaningful.

## Discussion

Recently, we have investigated the association between thyroid dysfunction and mortality [24]–[28]. While mortality is clearly increased, approximately 30% in hyperthyroidism, the underlying morbidities remain to be clearly defined [24]–[26]. In the present study, utilizing population-based Danish registers, we have evaluated the temporal relation between hyperthyroidism and different morbidities.

Disregarding temporality, we found a positive association between hyperthyroidism and CVD, LD, DM, and RD, which is in congruence with the literature [9], [23], [35]. These findings seem biologically plausible, since hyperthyroidism has been associated with hemodynamic changes [9], endothelial dysfunction [10] and coagulopathy [11]. Although hyperthyroidism does not affect the lung capacity directly [21], the hypermetabolic state and hyperthyroid myopathy affect cardiopulmonary performance [36]. In addition, autoimmune disorders like diabetes mellitus type 1, rheumatic diseases and Graveś disease seem to cluster in some patients [35]. However, many of the previously published associations between hyperthyroidism and morbidity are based on studies with inadequate power and as a consequence offer divergent findings. This hinders making any firm conclusions as for the temporal relation between hyperthyroidism and various morbidities [20].

Stratification for the period before and after the diagnosis of hyperthyroidism allows speculation with respect to the temporal relation between hyperthyroidism and other morbidities. We have shown that individuals with hyperthyroidism have an increased risk of being diagnosed with CVD, LD, DM and RD, prior to the diagnosis of hyperthyroidism. Following the diagnosis of hyperthyroidism, there was an increased risk of being diagnosed with CVD, LD, and DM. These findings could be interpreted as a direct association between CVD, LD and DM on the one hand and hyperthyroidism on the other hand, or *vice versa*. Moreover, it is important to point out that the treatment of the co-morbidity could also influence the observed association. This is best illustrated in CVD. Here the use of iodine containing anti-arhythmics (e.g. amiodarone) is a risk factor for the development of hyperthyroidism [14].

Another explanation for an increased risk of CVD, LD, and DM - both before and after the diagnosis of hyperthyroidism - could be non-thyroidal illness [15] or confounding by indication [16]. The phenomenon that the detection of one disease leads to higher rates of detection of other, and not necessarily related, diseases is well described [16]. In order to minimize this so-called Berksońs bias, we applied censoring of diagnoses made 365 days before or after the diagnosis of hyperthyroidism. This only affected the risk of CVD after the diagnosis of hyperthyroidism, which in contrast to the analysis without censoring, was not increased significantly. Although atrial fibrillation or heart failure, both frequent cardiac complications [12], most likely remit after restoration of euthyroidism [12], the association between hyperthyroidism and CVD could partly be due to confounding by indication. Whether this also applies for the other disease groups remains unclarified. However, the fact that the 365 days censoring of registration of co-morbidity did not significantly change our results argues against such an effect.

The pattern of an increased risk of CVD, LD, and DM both before and after the diagnosis of hyperthyroidism could also be explained by the presence of shared environmental or genetic factors associated with CVD, LD, DM, and hyperthyroidism. As an example, smoking is a risk factor for hyperthyroidism [4], but also for CVD and LD [37]. Furthermore, both hyperthyroidism [17], CVD [18], LD [38], and DM [39] show familial aggregation. If these conditions cross-trait, the finding of an increased risk of morbidity associated with hyperthyroidism could be due to genetic confounding. Based on our findings within MZ twin pairs discordant for hyperthyroidism, we cannot completely rule out this possibility. Unfortunately, despite the population-based study design, there were too few twin pairs to allow stratification with respect to type of hyperthyroidism (GD or TNG) and co-morbidity.

While both GD and TNG are associated with an increased mortality, they differ with respect to the cause of death [26]. Cardiovascular mortality is significantly increased in GD when compared to TNG [26]. In addition, GD and TNG have been shown to differ with respect to cardiac morbidity [12]. Cardiac valve degeneration, as an example, mostly occurs in GD and other autoimmune thyroid disorders [12]. In contrast, TNG more frequently than GD, has been found associated with cardiac arrhythmias and heart failure [12]. However, this could obviously pertain to the higher age and consequently higher co-morbidity in TNG individuals [12]. In line with this view, the findings of the present study, where cases and controls are age matched, indicate no difference between GD and TNG, neither with respect to CVD nor with respect to DM and LD, before or after the diagnosis of hyperthyroidism. Still, the exact pathophysiological mechanisms related to the differences in cardiovascular mortality in GD and TNG require further investigations.

The strengths of our study include a large sample size, ascertainment of participants from nationwide population-based registers, use of standardized and validated procedures for evaluating the degree of morbidity [33], [40], and a long observation period. The possibility of stratifying for the time period before and after the diagnosis of hyperthyroidism is unique to our study, and allows speculation with respect to causality between hyperthyroidism and any outcome of interest, such as morbidity. Importantly, the fact that patients from both DNPR and DNPrR, and consequently both patients from a hospital setting – whether out- or in-patient - and primary care are accounted for, minimizes the risk of selection bias. On the other hand, the lack of information regarding the type of therapy, effect of treatment, the inability to validate the DNPrR with respect to hyperthyroidism, and lack of biochemical data are weaknesses in the present study. Additionally, our definition of disease groups may be too simplified, and consequently our evaluation of morbidity may be incomplete.

In conclusion, we demonstrate a significantly increased burden of morbidity, both before and after the diagnosis of hyperthyroidism. However, we cannot completely exclude the influence of genetic confounding.
